# In situ Electrochemical Evaluation of the Interaction of dsDNA with the Proteasome Inhibitor Anticancer Drug Bortezomib

**DOI:** 10.3390/molecules28073277

**Published:** 2023-04-06

**Authors:** Mihaela-Cristina Bunea, Teodor Adrian Enache, Victor Constantin Diculescu

**Affiliations:** National Institute of Materials Physics, Atomistilor 405A, 077125 Magurele, Romania

**Keywords:** bortezomib, redox products, DNA, interaction, dsDNA electrochemical biosensor

## Abstract

Bortezomib is an inhibitor of proteasomes and an anti-cancer drug. Although bortezomib is considered a safe drug, as confirmed by cytotoxicity assays, recent reports highlighted the possibility of interaction between bortezomib and cellular components, with detrimental long-term effects. The evaluation of the interaction between bortezomib and dsDNA was investigated in bulk solution and using a dsDNA electrochemical biosensor. The binding of bortezomib to dsDNA involved its electroactive centers and led to small morphological modifications in the dsDNA double helix, which were electrochemically identified through changes in the guanine and adenine residue oxidation peaks and confirmed by electrophoretic and spectrophotometric measurements. The redox product of bortezomib amino group oxidation was electrochemically generated in situ on the surface of the dsDNA electrochemical biosensor. The redox product of bortezomib was shown to interact primarily with guanine residues, preventing their oxidation and leading to the formation of bortezomib–guanine adducts, which was confirmed by control experiments with polyhomonucleotides electrochemical biosensors and mass spectrometry. An interaction mechanism between dsDNA and bortezomib is proposed, and the formation of the bortezomib redox product–guanine adduct explained.

## 1. Introduction

Proteasome inhibitors are a new generation of drugs used for the treatment of numerous types of cancers due to their capacity to induce the degradation of tumors [1]. Bortezomib (BTZ), [(1*R*)-3-methyl-1-[[(2*S*)-1-oxo-3-phenyl-2-[(pyrazinylcarbonyl) amino]propyl]amino]butyl] boronic acid, is the first proteasome inhibitor that was approved in May 2003 by the FDA for the treatment of hematological disorders, especially for multiple myeloma [2,3]. Since proteasomes are overexpressed during the disease phase, it has been shown that treatment with BTZ stimulates the degradation of anti-apoptotic proteins while preventing the degradation of pro-apoptotic ones [4,5,6,7]. Apart from multiple myeloma, investigations evaluated this drug both in vitro and in vivo against other types of disease including pancreatic, prostatic or ovarian cancers, glioma, and squamous cell carcinoma, and the reported results highlighted an important anticancer activity [8,9,10,11]. Another important property reported in the specialized literature regarding BTZ is the possibility to increase the sensitivity of chemo-resistant multiple myeloma cell lines to chemotherapeutic agents such as melphalan and doxorubicin [12]. However, as for most of the anticancer drugs, side effects also occur during treatment with BTZ. Serious side effects were observed mainly on neural cells [13], and some other adverse events are gastrointestinal symptoms, including nausea and vomiting, diarrhea, and constipation, fatigue, thrombocytopenia, and sensory neuropathy [12].

Due to its potential benefits, the characterization of BTZ represents an important issue and has led to the publication of numerous analytical studies [13,14,15,16]. Among these, studies on the charge-transfer reaction mechanisms of BTZ have been reported in the literature, investigating BTZ voltammetric behavior [17], an important topic since BTZ may participate in vivo in several redox reactions. On the other hand, the evaluation of DNA oxidative damage and the mechanisms of BTZ interaction with different molecules represents an important issue in the development of more efficient anticancer pharmaceutics [18,19,20,21]. The dsDNA electrochemical biosensor consists of dsDNA immobilized on the surface of an electrochemical transducer. The most used type of electrochemical transducer is the glassy carbon electrode due to its unique properties such us large potential window and ease in surface modification [18,22,23]. These properties enable the identification of the DNA oxidation peaks of purine (guanine and adenine) and pyrimidine (thymine and cytosine) bases and of their oxidation products (8-oxo-guanine and 2,8-dihydroxyadenine), which at the same time represents biological markers of oxidative stress, allowing the monitoring of potential DNA damages [24,25,26] induced by different hazard chemicals, including pharmaceutical compounds.

Electrochemical studies performed on different drugs such as immunosuppressants, anti-cancer molecules, antibiotics, or sedatives, in general, and on BTZ, in particular, have the potential to provide valuable insights into the understanding of the mechanisms by which these compounds exert their biological activity but also useful information for the evaluation of their pharmacokinetics [27,28,29,30,31,32]. In this context, the main purpose of this study was the evaluation of BTZ–dsDNA interaction. With this scope, two different electrochemical procedures were used. First, electrochemical investigations were performed in mixed BZT–dsDNA solutions where DNA flexibility was an important issue in understanding the initial steps of the interaction mechanism. Then, the dsDNA electrochemical biosensor was employed, which allowed monitoring in situ and in real time the interaction of redox products of BTZ with the immobilized dsDNA. The electrochemical data were confirmed by other analytical techniques such as UV–Vis spectrophotometry, gel electrophoresis, and mass spectrometry.

## 2. Results and Discussion

**Figure 1:**
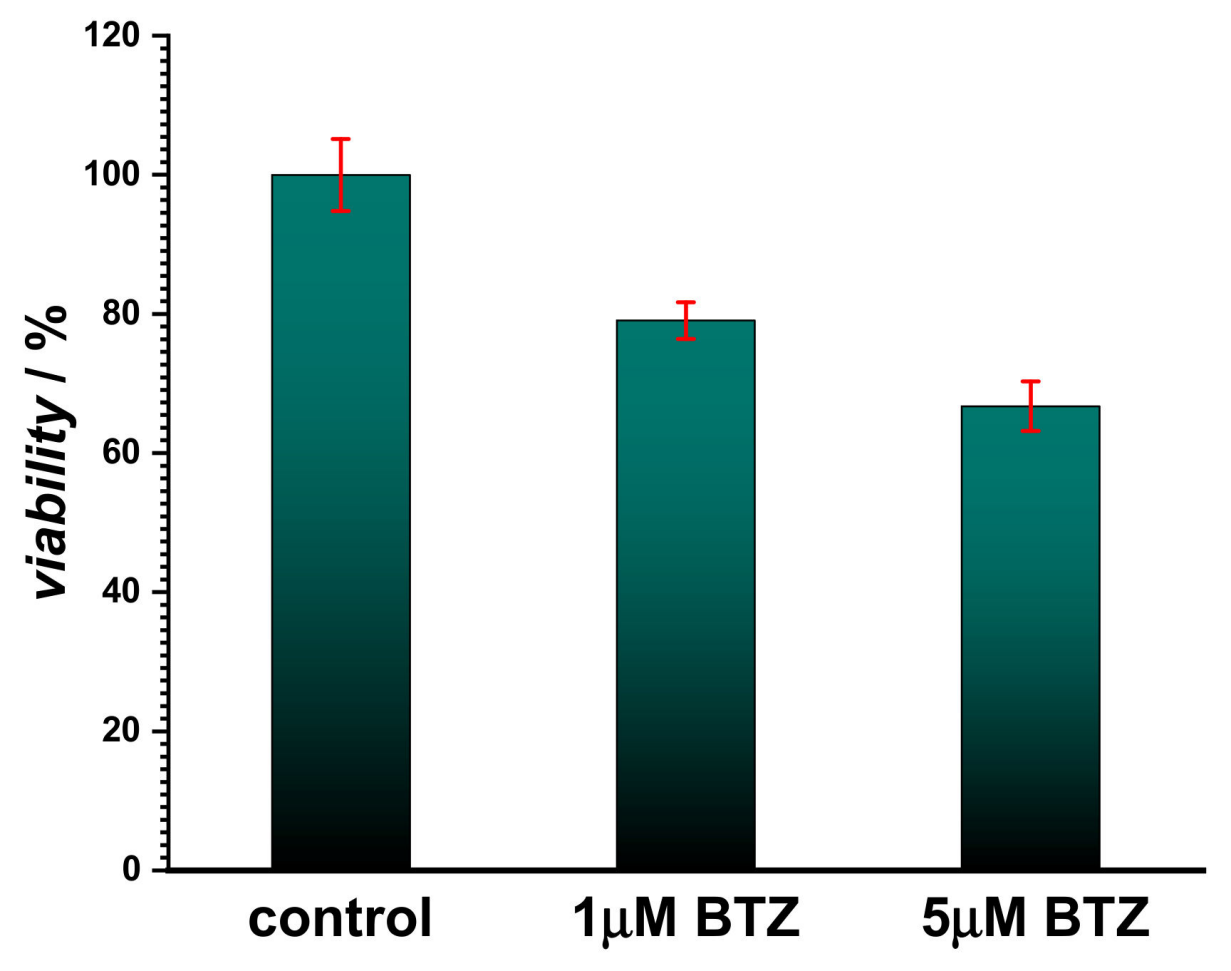
Cell viability. MTS test with the L929 cells line.

Initially, the assessment of BTZ cytotoxicity was investigated using the fibroblast cell line L929 and the MTS assay (Figure 1). The results showed that for concentrations below 1 µM, BTZ did not induce cell death, and cell viability was found to be over 80%. However, a slight toxicity was observed for concentrations higher than 5 µM, since cell viability decreased by approx. 30% when compared to the control, indicating that less than 70% of the cells remained metabolically active. Although BTZ is considered a safe drug and the cytotoxicity assay confirmed this, recent reports highlighted the possibility of interaction between BTZ and cellular components [13,16], with detrimental long-term effects.

Since DNA is one of the most important biomolecules in a living cell, the main objective of this study was to assess and understand BTZ interaction with dsDNA using two different methods: voltametric analysis with a GCE in mixed BTZ–DNA solutions and with the dsDNA electrochemical biosensor, which can provide fast and real-time results. The electrochemical results were correlated with the spectrophotometric, gel electrophoresis, and mass spectrometry results, and a possible interaction mechanism between BTZ redox products and DNA is proposed.

### 2.1. Voltammetric Behaviour of Bortezomib and dsDNA

**Figure 2:**
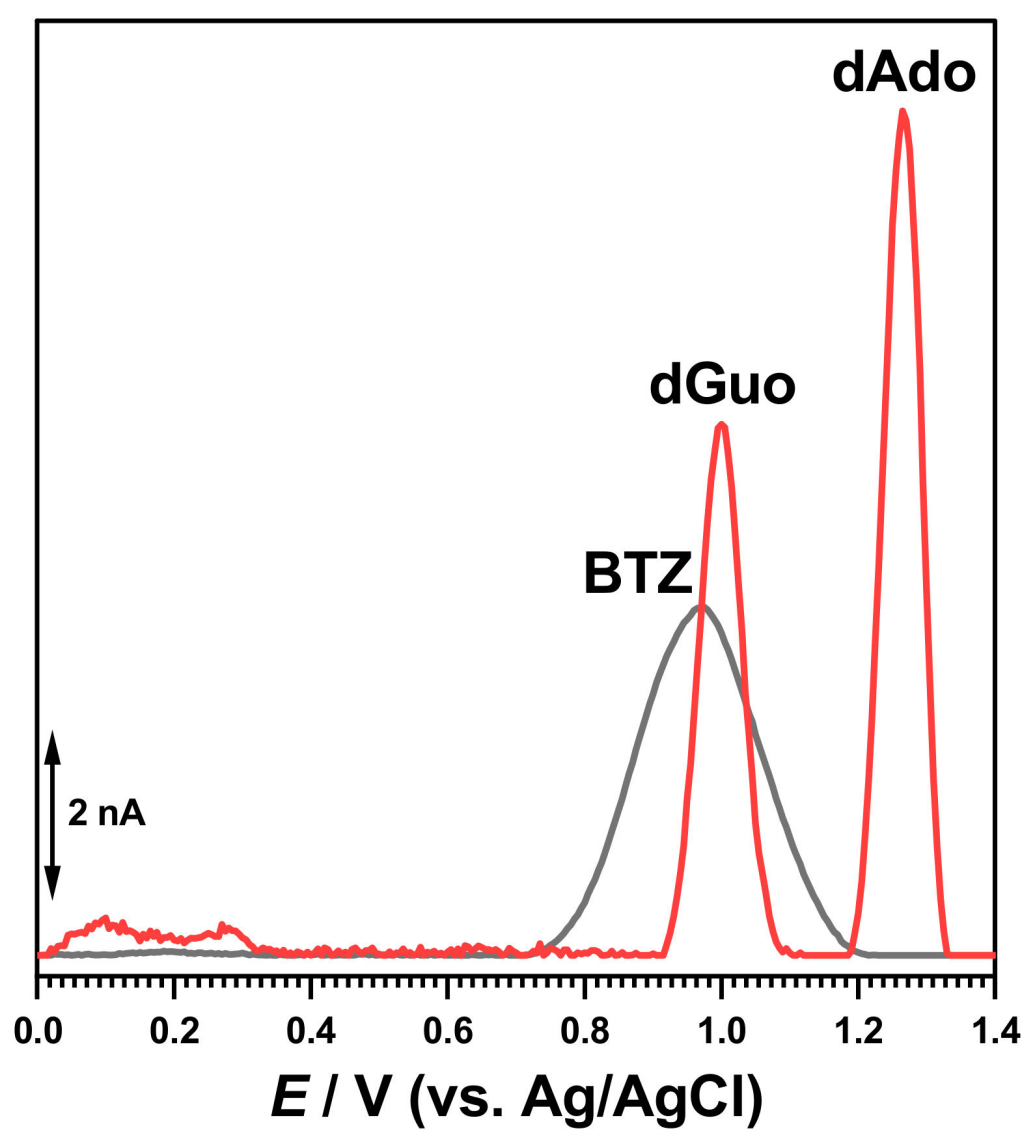
DP voltammograms, baseline-corrected, of solutions of 50 µg mL^−1^ of dsDNA (red curve) and 30 µM BTZ (black curve) at pH = 4.5 in 0.1 M acetate buffer.

The electrochemical characterization of BTZ and dsDNA in 0.1 M acetate buffer, pH = 4.5, was carried out as a control (Figure 2). The DP voltammogram recorded in a solution of 30 μM BTZ showed one charge-transfer reaction at *E*_p_ = +0.94 V, due to one electron and one proton transfer from the amino group close to the pyrazine ring, as previously reported [17]. On the second DP voltammogram obtained in the same conditions without cleaning the electrode surface, the BTZ peak current decreased due to the adsorption of the BTZ oxidation products on the GCE surface.

The DP voltammograms recorded in a solution of 50 µg mL^−1^ of dsDNA presented two consecutive peaks due to purine bases within the DNA helix, i.e., dGuo of guanosine residues at *E*_p_ = +1.01 V and dAdo of adenosine residues at *E*_p_ = +1.28 V, as shown in Figure 2.

### 2.2. Evaluation of Bortezomib–dsDNA Interaction in Mixed Solutions

#### 2.2.1. Voltammetry

The electrochemical study of BTZ–dsDNA interaction was conducted by recording DP voltammograms in mixed solutions after different incubation periods by comparing the changes on the dsDNA oxidation peaks, dGuo and dAdo, in the presence of BTZ and by monitoring the occurrence of the oxidation peaks of Gua, Ade, and the guanine and/or adenine oxidation products 8-oxo guanine (8-oxoGua) and 2,8-dihydroxy adenine (2,8-oxoAde) [18].

**Figure 3:**
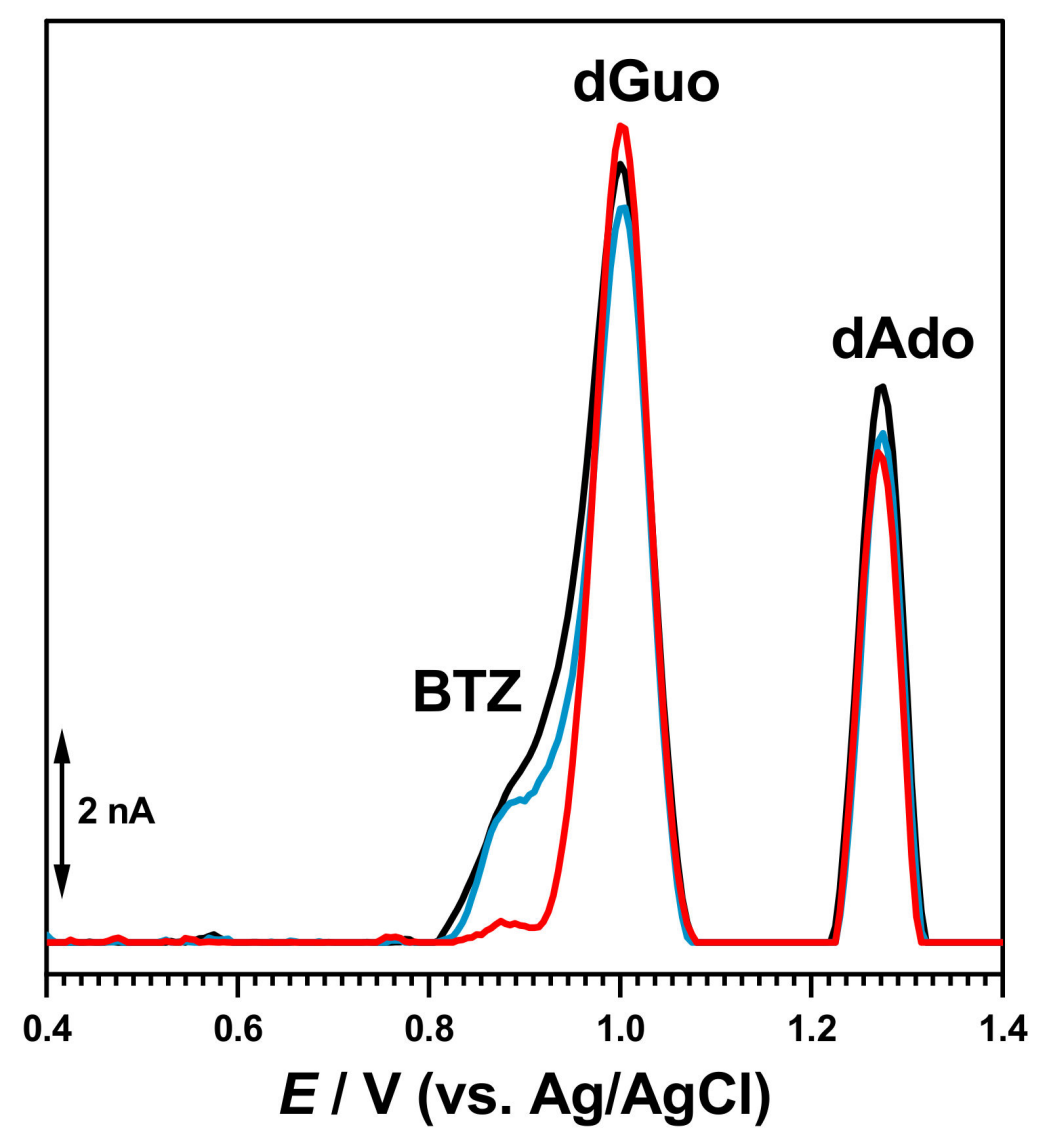
DP voltammograms, baseline-corrected, of solutions of 50 µg mL^−1^ of dsDNA and 30 µM BTZ incubated after 0.00 (black curve), 0.50 (blue curve), and 1.00 h (red curve) of incubation in 0.1 M acetate buffer, pH = 4.5.

Initial experiments were performed after incubation of 30 µM BTZ and 50 µg mL^−1^ DNA solutions for different times. After each measurement, the GCE surface was cleaned and rinsed with deionized water to remove all oxidation products adsorbed on the electrode surface. The DP voltammogram recorded at 0 h incubation time showed the occurrence of the oxidation peak of free/uncomplexed BTZ molecules superimposed with that of dGuo of dsDNA and the dAdo peak at higher positive potential values (Figure 3).

For increased incubation times (Figure 3), the oxidation peak current of free/uncomplexed BTZ molecules progressively decreased in a time-dependent manner, demonstrating that less BTZ molecules were available for oxidation and that the BTZ electroactive centers were involved in the interaction. At the same time, small modifications of the dGuo and dAdo oxidation peaks were observed, in agreement with conformational modification of the dsDNA double helix.

#### 2.2.2. UV–Vis Spectrophotometry

**Figure 4:**
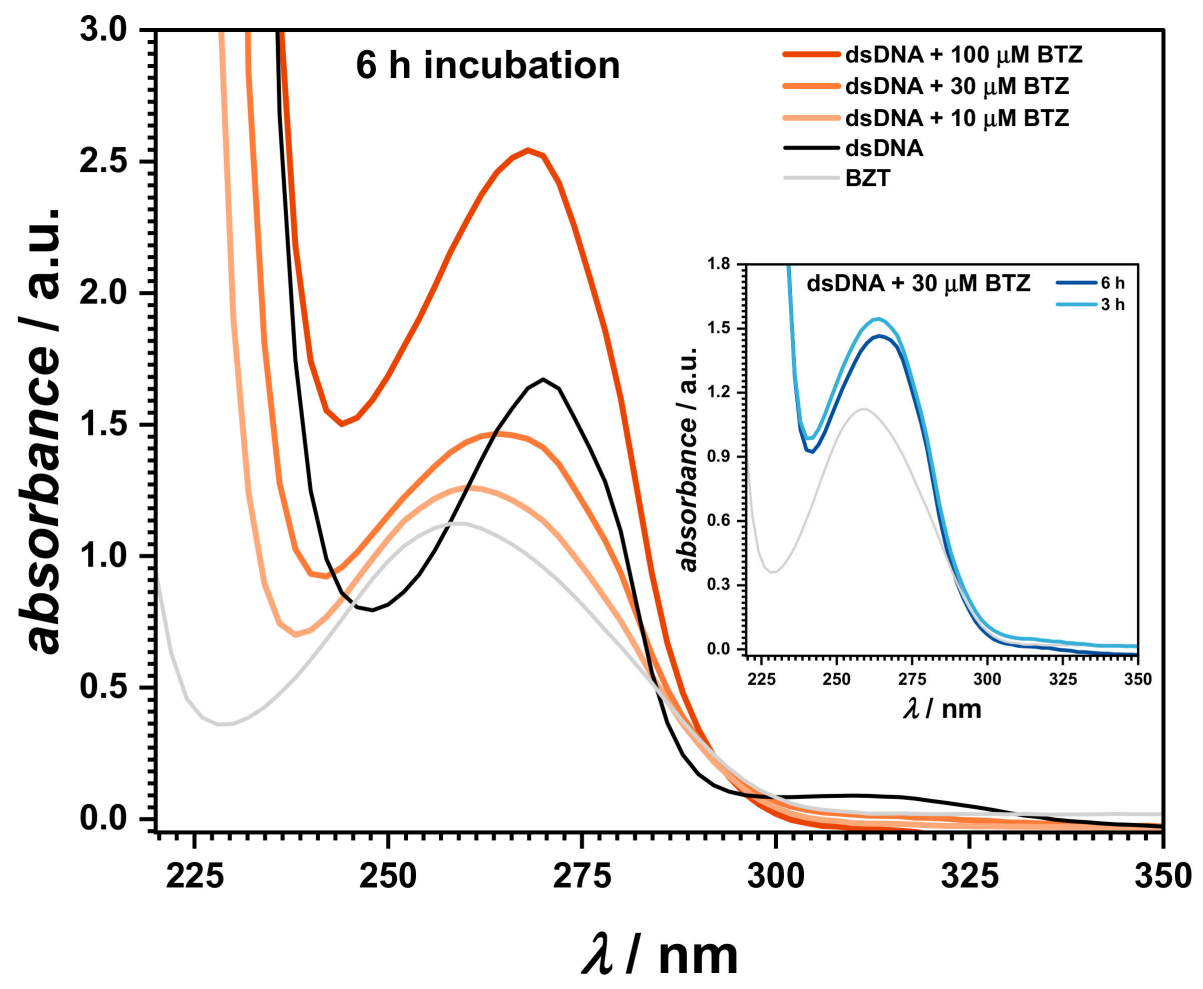
UV spectra of a 50 μg mL^−1^ dsDNA solution (grey curve), a 100 μM BTZ solution (black curve), and mixt solutions of 10, 30, and 100 μM BTZ incubated during 6 h with 50 μg mL^−1^ of dsDNA at pH = 4.5 in 0.1 M acetate buffer. The inset shows the spectra recorded after the incubation of the 50 μg mL^−1^ dsDNA solution with the 30 μM BTZ solution during 3 and 6 h.

UV–Vis spectrophotometry was also used to investigate the interaction of BTZ with dsDNA, as shown in Figure 4. The spectra were recorded in solutions containing 30 μM BTZ and 50 μg mL^−1^ of dsDNA at pH = 4.5 in 0.1 M acetate buffer, at different incubation times (Figure 4, inset). Control samples consisting of 100 μM BTZ and 50 μg mL^−1^ of dsDNA were also prepared and measured in the same conditions.

The UV spectra of the control samples showed an absorption band at 259 nm for dsDNA, while BTZ presented one main absorption band at 272 nm, followed by a smaller peak at 318 nm, attributed to the pyrazine ring [32].

UV spectra were also recorded for mixt solutions of 50 μg mL^−1^ of dsDNA with different concentrations of BTZ. When increasing the BTZ concentrations, a red shift of the DNA absorption band of about 2 nm, to 280.6 nm, was observed. At the same time, the BTZ band at 318 nm disappeared, and an isosbestic point occurred at 293 nm, proving the binding of BTZ to the DNA strands and the formation of a BTZ–dsDNA complex. The time dependence of the BTZ–dsDNA interaction was investigated, and the spectra recorded after 3 and 6 h of incubation showed a time-dependent increase of the absorbance at 280.6 nm, in agreement with the slight unwinding/relaxation of the dsDNA structure.

#### 2.2.3. Gel Electrophoresis

**Figure 5:**
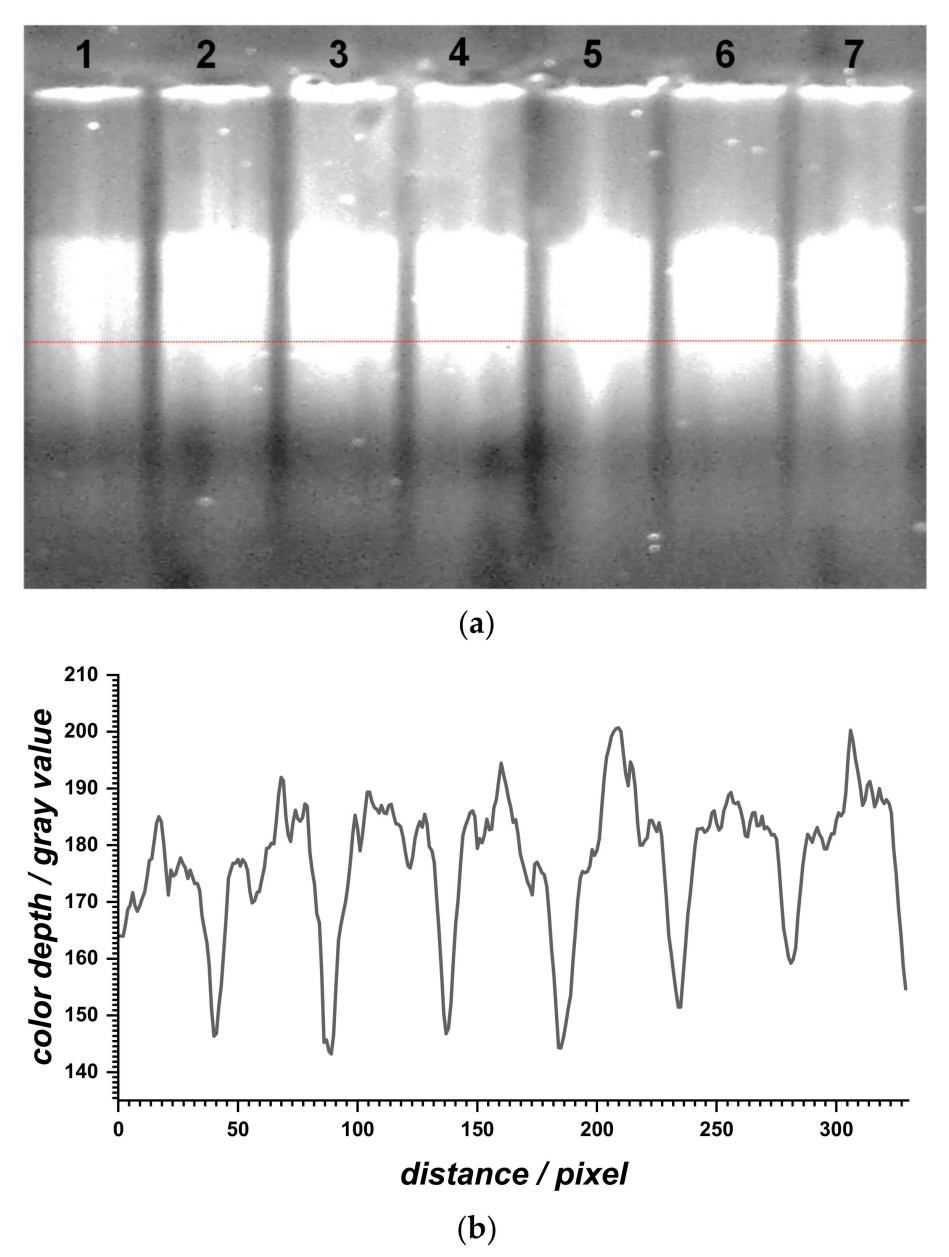
(**a**) Gel electrophoresis of the 50 µg mL^−1^ dsDNA control solution (lane 1) and after its incubation for 6 h in 0.1 M acetate buffer, pH = 4.5, with BTZ: 10 μM (lane 2), 25 μM (lane 3), 50 μM (lane 4), 75 μM (lane 5), 100 μM (lane 6), and 150 μM (lane 7); the red line represents the front of the migration profile. (**b**) Electrophoretic bands’ intensity at the front of the migration profile.

Agarose gel electrophoresis was performed to evaluate the relative migration profile of the dsDNA samples before and after dsDNA interaction with BTZ at different concentrations (Figure 5). The dsDNA sample (lane 1) showed a band due to different-size, long, and three-dimensional fragments present in the calf thymus dsDNA sample. When increasing the BTZ concentration (lanes 2 to 7), a slight increase in the DNA migration relative to the control sample (lane 1) was observed and, at the same time, the corresponding bands became more intense.

In general, the migration distances of DNA fragments vary as a function of the number of base pairs but also due to the accelerated mobility of unwound double helixes. BTZ binding to dsDNA led to the formation of a BTZ–dsDNA complex, with a slight unwinding/relaxation of the dsDNA structure, which is in agreement with the increase of its migration.

### 2.3. Evaluation of Bortezomib–dsDNA Interaction by the dsDNA Electrochemical Biosensor

During the voltammetric experiments with solutions of dsDNA and BTZ, the formation of an incomplete film of co-adsorbed free dsDNA, free BTZ, and ds-DNA-BTZ complexes on the surface of the GCE occurred. In order to avoid the complications of diffusion processes, the interaction mechanism of BTZ with dsDNA was investigated using the dsDNA electrochemical biosensor. The main advantage of this methodology is that the non-specific adsorption of BTZ on the electrode surface is eliminated due to the complete coverage of the GCE surface with the dsDNA film.

**Figure 6:**
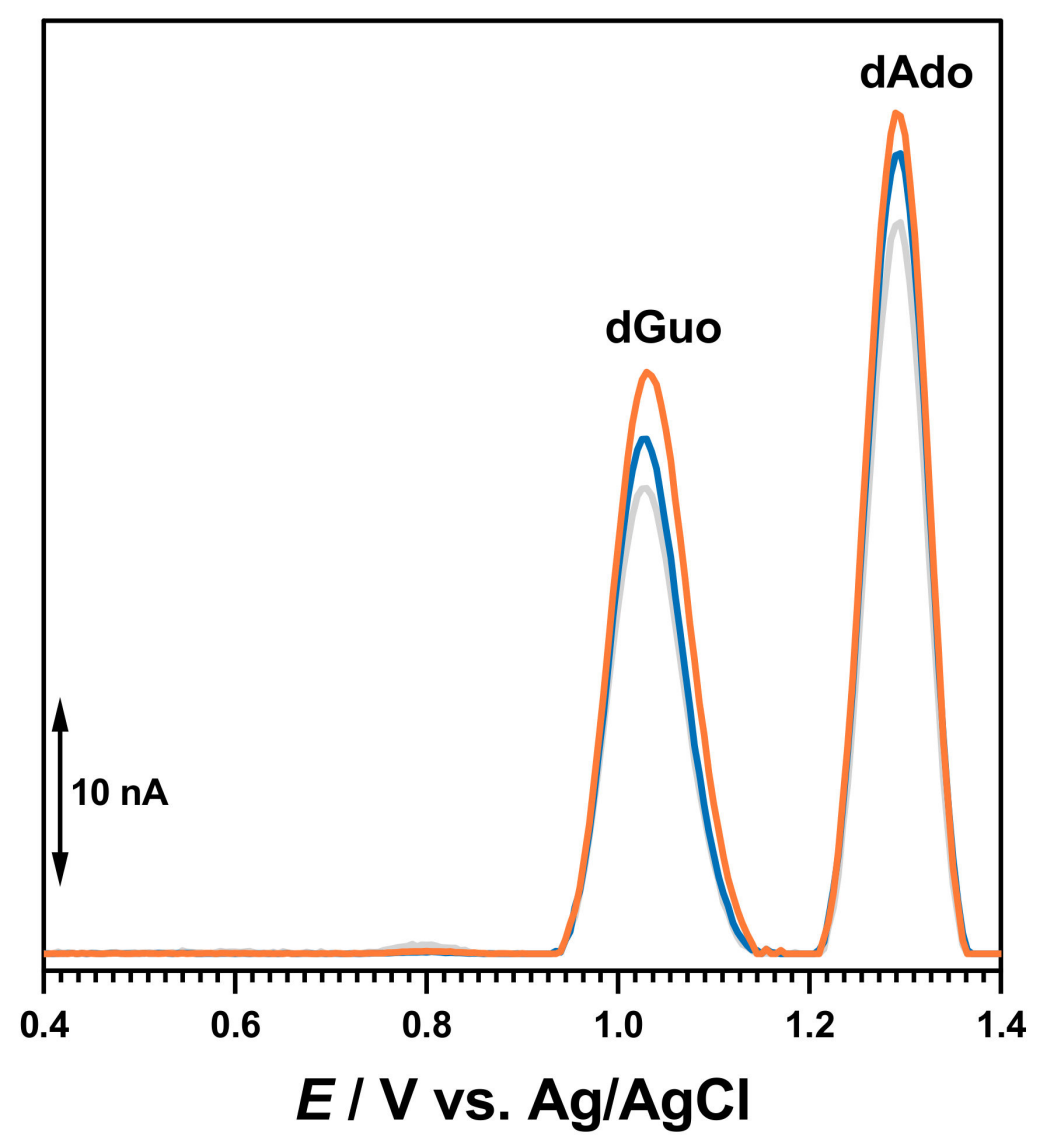
DP voltammograms, baseline-corrected, in 0.1 M acetate buffer, pH = 4.5, in the presence of the dsDNA biosensor before (grey curve) and after incubation for 10 (blue curve) and 20 min (orange curve) in 100 µM BTZ.

The interaction of BTZ with dsDNA was initially evaluated after the incubation of the dsDNA electrochemical biosensor during different times in solutions containing different concentrations of BTZ, with no applied conditioning potential. The DP voltammogram recorded with the dsDNA electrochemical biosensor in buffer showed both dGuo and dAdo oxidation peaks. In a new experiment, a newly prepared dsDN-electrochemical biosensor was incubated during 10 min in the 100 μM BTZ solution (Figure 6).

Then, the dsDNA electrochemical biosensor was washed with deionized water to remove unbound BTZ molecules and transferred to the electrochemical cell containing only 0.1 M acetate buffer, pH = 4.5. The DP voltammograms recorded in these conditions showed only the dGuo and dAdo peaks, which increased with the incubation time in agreement with dsDNA conformational modifications and the unwinding of the double helix. No BTZ oxidation appeared, showing that the electroactive centers were involved in the interaction with DNA.

### 2.4. In Situ Evaluation of Bortezomib Redox Product–dsDNA Interactions

#### 2.4.1. dsDNA Electrochemical Biosensor

The interaction of the BTZ oxidation products with dsDNA was also evaluated after the incubation of the dsDNA electrochemical biosensor under a +0.90 V applied potential. This potential value was chosen so that the BTZ molecules that diffused from the solution toward the dsDNA electrochemical biosensor surface during the incubation in the BTZ solution were immediately oxidized. It is important to mention that the application of the +0.90 V potential did not oxidize the immobilized dsDNA, since the dGuo peak occurred at higher potential values. Thus, the dsDNA electrochemical biosensors was capable to provide in situ and in real time information on the interaction between the BTZ redox products and DNA.

**Figure 7:**
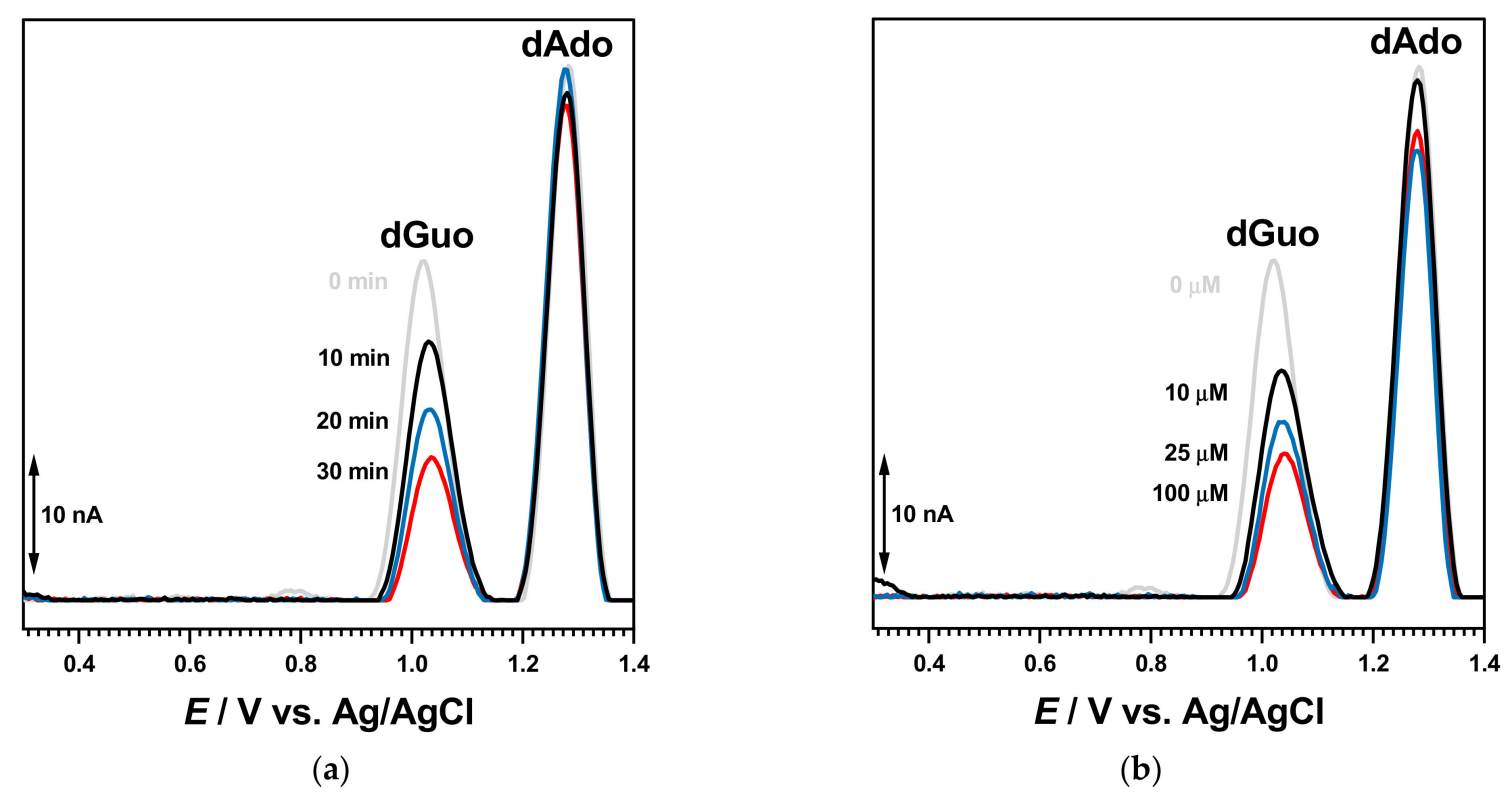
DP voltammograms, baseline-corrected, in 0.1 M acetate buffer, pH = 4.5, with the dsDNA-biosensor after incubation at +0.90 V: (**a**) with 100 µM BTZ during different times, (**b**) during 30 min with different concentrations of BTZ.

In these conditions, the dsDNA electrochemical biosensor was incubated at +0.90 V during 10 min in the 100 μM BTZ solution. Then, the dsDNA electrochemical biosensor was washed with deionized water in order to remove the unbound BTZ molecules. The DP voltammogram recorded in 0.1 M acetate buffer, pH = 4.5 (Figure 7a) showed the decreased of the dGuo oxidation peak and a positive shift of its potential value when compared to the control experiment. When increasing the incubation time at +0.90 V, a progressive time-dependent decrease of the dGuo oxidation peak occurred due to the interaction between the guanine residues in the dsDNA and the in situ electrogenerated redox product of BTZ. It is important to note that each experiment was carried out with a newly prepared dsDNA electrochemical biosensor.

A similar experiment was carried out with newly prepared dsDNA electrochemical biosensors, which were incubated for 30 min in solutions with different BTZ concentrations, as shown in Figure 7b. The voltammograms recorded in the buffer showed a similar behavior, and the dGuo peak decreased in a time-dependent manner with the increasing BTZ concentration.

**Figure 8:**
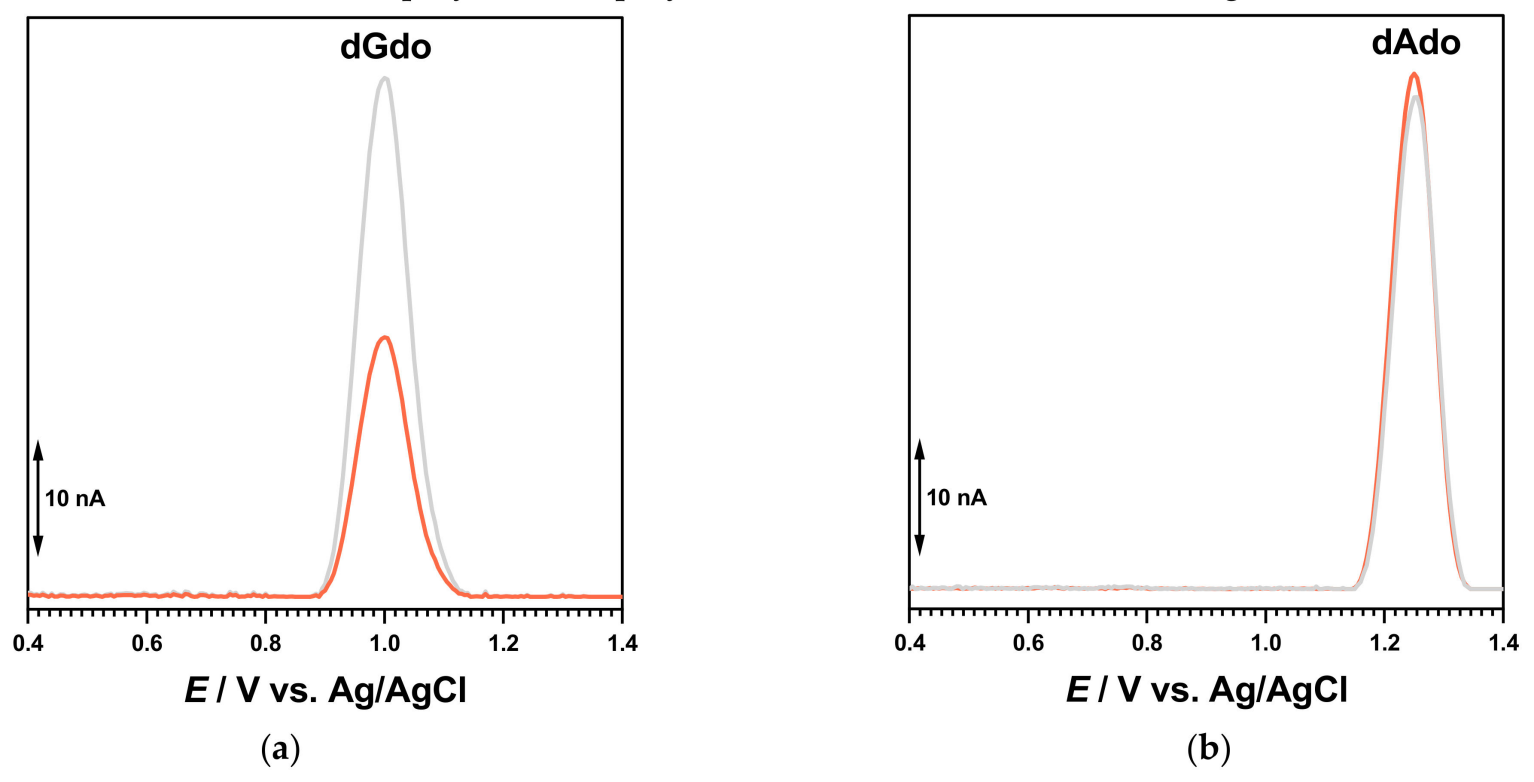
DP voltammograms, baseline-corrected, in 0.1 M acetate buffer, pH = 4.5, with the (**a**) poly[G]-biosensor and (**b**) poly[A]-biosensor after incubation at +0.90 V in buffer (grey curve) or in a solution of 100 µM BTZ (orange curve) during 30 min.

These experiments indicated a clear interaction between the in situ electrogenerated redox product of BTZ oxidation and the guanine residues. In order to verify these interactions, experiments were also carried out using purine homopolynucleotide single-stranded poly[A]- and poly[G]-electrochemical biosensors (Figure 8).

The DP voltammogram in 0.1 M acetate buffer, pH = 4.5, with the poly[G]-electrochemical biosensor showed one anodic peak at +1.01 V, corresponding to the oxidation of dGuo. A newly prepared poly[G]-electrochemical biosensor was incubated for 30 min in 100 µM BTZ at an applied potential of +0.90 V. The guanine residue oxidation peak decreased, in agreement with the interaction between guanine residues in the dsDNA and the in situ electrogenerated redox product of BTZ. In contrast, similar experiments carried out with the poly[A]-electrochemical biosensor did not show any variation of the dAdo peak, proving the affinity of the BTZ redox product for the guanine residues of DNA.

#### 2.4.2. Mass Spectrometry

**Figure 9:**
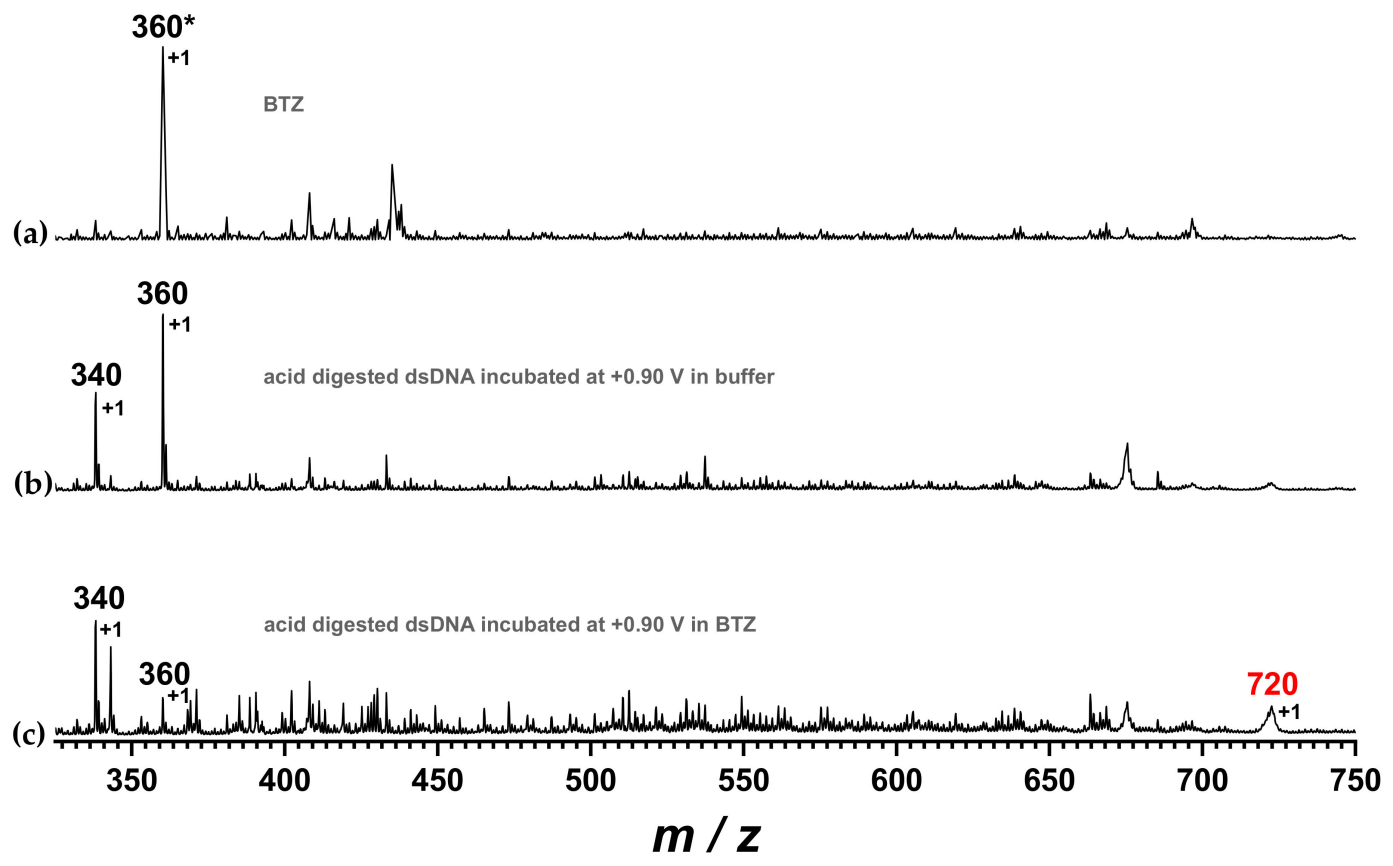
MS spectra of (**a**) BTZ (marked with *) and acid-digested dsDNA after incubation during 30 min at +0.90 V in (**b**) 0.1 M acetate buffer, pH = 4.5 and (**c**) a BTZ solution.

Drugs interact with DNA through several mechanisms including adduct formation, apart from electrostatic binding or intercalation. The experiments above-described showed the decrease of the dGuo oxidation peak upon incubation of the dsDNA electrochemical biosensor at +0.90 V in the BTZ solution, in agreement with an adduct formation between the guanine residues and the BTZ oxidation product. The possibility of adduct formation between DNA bases and a BTZ redox product was investigated using mass spectrometry, as shown in Figure 9. For this investigation, the DNA film was removed from the surface of the biosensor after incubation in pure buffer or after interaction with BZT at +0.90 V and then subjected to acidic digestion, as described in Section 3.5.

The MS spectrum of BTZ showed one main signal with *m*/*z* value at approx. 360. The MS spectrum of the DNA after incubation at +0.90 V in 0.1 M acetate buffer, pH = 4.5, without interaction with the BTZ redox product, showed signals corresponding to free DNA nucleotides, i.e., adenosine monophosphate and guanosine monophosphate, with *m*/*z* values of approx. 340 and 360, respectively. Nonetheless, the MS spectrum of the dsDNA sample upon incubation with BTZ at +0.90 V showed the decrease of the guanosine monophosphate signal at 360 a.m.u. and the occurrence of a new signal with *m*/*z* value of 720 a.m.u., which corresponds to the mass of the BTZ–GMP adduct.

### 2.5. Interaction Mechanism of Bortezomib and Its Redox Product with DNA

The experiments carried out in solutions incubated for different times and with the dsDNA electrochemical biosensor showed that BTZ binding to dsDNA involved small conformational modifications of the double helix that could be detected through changes in the dGuo and dAdo oxidation peaks (Figure 3 and Figure 6), which were also confirmed by spectrophotometric (Figure 4) and electrophoretic (Figure 5) measurements. At the same time, the electrochemical experiments in solutions incubated for different times (Figure 3) showed that the BTZ oxidation peaks decreased progressively in a time-dependent manner, indicating that the interaction with dsDNA involved the electroactive centers of BTZ, i.e., the amino group in the proximity of the pyrazine ring. Similarly, UV–Vis spectrophotometry (Figure 4) showed also the disappearance of the adsorption band at 318 nm, attributed to the same moiety. However, no oxidative damage to DNA was observed, in agreement with the cytotoxicity test shown in Figure 1.

**Scheme 1:**
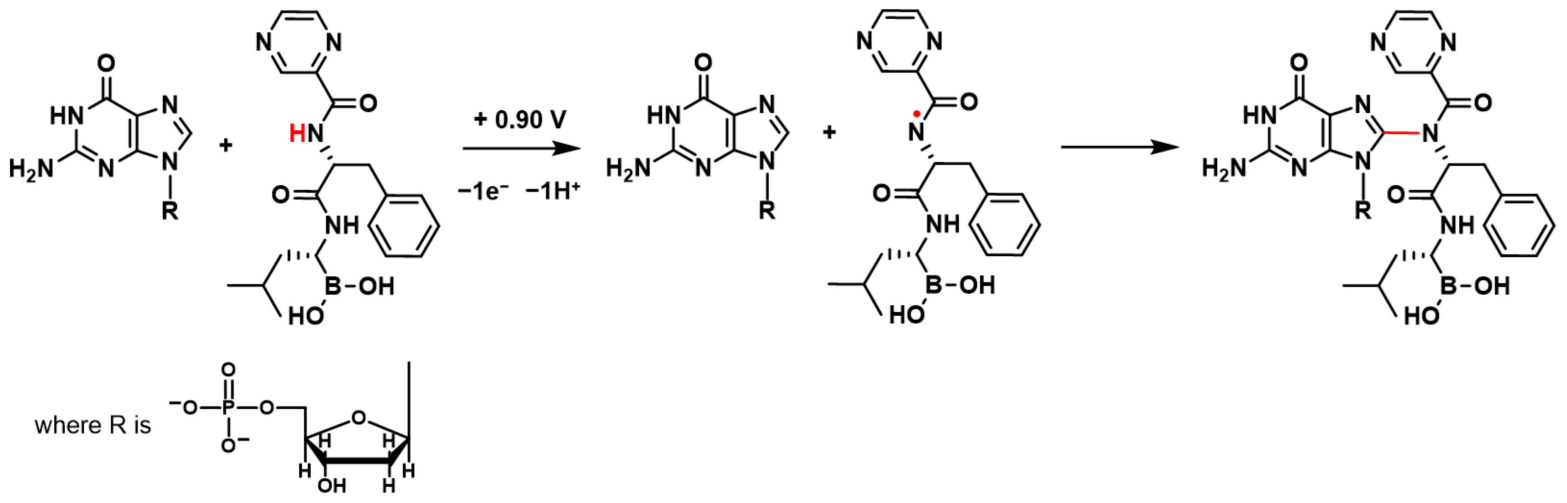
Proposed mechanism for the interaction between the guanine residues of DNA and the BTZ redox product.

BTZ oxidation involves the transfer of electrons and protons from the amino group in the proximity of the pyrazine ring, leading to the formation of a radical cation, which undergoes hydrolysis [17] (Scheme 1). Such kind of radicals from the amine-containing compound are well known to interact with and covalently attach to dsDNA, leading to the formation of different DNA–base adducts [26,27].

When the +0.90 V conditioning potential was applied to the dsDNA electrochemical biosensor in the BTZ solution, the BTZ redox product formed on the dsDNA electrochemical biosensor surface and immediately interacted with dsDNA guanine residues (Scheme 1). The BTZ redox product–guanine adduct involved the guanine electroactive center at position C8 (Scheme 1), explaining the dGua oxidation peak decrease observed in Figure 7 and Figure 8. Nonetheless, the formation of the BTZ redox product–guanine adduct was also demonstrated by MS spectrometry, where the signal at *m*/*z* = 720 a.m.u. corresponds to the mass of the BTZ–guanosine monophosphate adduct.

## 3. Materials and Methods

### 3.1. Reagents and Solutions

Bortezomib (BTZ) was from Selleck Chemicals, sodium salt double-stranded DNA (dsDNA) from calf thymus, polyadenylic acid (poly[A]), and polyguanylic acid potassium salt (poly[G]) from Sigma-Aldrich (St. Louis, MO, USA) and were used without further purification. For the in vitro cytotoxicity assay, the fibroblast L929 cell line from ATCC (Manassas, VA, USA) was used. Other materials used were: Dulbecco’s Modified Eagle Medium (DMEM, Waltham, MA, USA) and MTS assay kit from Sigma-Aldrich, phosphate buffer saline (PBS) from Thermo Fisher Scientific (Waltham, MA, USA), fetal bovine serum (FBS) from Thermo Fisher Scientific, trypsin from Lonza Bioscience Solutions (St. Bend, OR, USA), and antibiotics from Biological Industries (Cromwell, CT, USA).

The stock solution of 500 µM BTZ was prepared in a DMSO/deionized water mixture (1% *v*/*v*) and kept at 4 °C. The solutions of different concentrations of BTZ were prepared by dilution of the appropriate quantity directly in the supporting electrolyte.

The electrolyte solution of 0.1 M acetate buffer, pH = 4.5, was prepared with analytical-grade reagents and purified water from a Millipore Milli-Q system (conductivity less than 0.1 μS cm^−1^).

The stock solutions of 500 µg mL^−1^ of dsDNA, poly[A] and poly[G] were prepared in purified water from the Milli-Q system and diluted to the desired concentration prior to use.

All experiments were performed at room temperature (25 ± 1 °C).

### 3.2. Voltammetry

The voltammetric experiments were performed using a Ivium potentiostat running IviumSoft version 4.977 (Ivium Technologies, Eindhoven, The Netherlands), a glassy carbon working electrode (d = 1.6 mm), a Pt wire counter, and a Ag/AgCl (3 M KCl) reference electrode in a one-compartment electrochemical cell (eDAQ Europe) containing 0.5 mL of electrolyte. The GCE surfaced was renewed before each experiment using diamond spray (particle size 1 μm) on a microcloth pad, rinsed with Milli-Q water, and electrochemically pre-treated by recording various DP voltammograms in a buffer-supporting electrolyte until a steady-state baseline voltammogram was obtained.

Differential-pulse (DP) voltammetry was recorded with a pulse amplitude of 50 mV, a pulse width of 100 ms, and a scan rate of 5 mV s^−1^. The DP voltammograms presented were previously background-subtracted and baseline-corrected trough the IVIUM soft program tools. The mathematical treatment used leads to a 10% decrease in the height of the peak but it was used for all the experimental voltammograms for a better and clearer identification of the peaks. The values of the peak current presented in all graphs were determined from the original untreated voltammograms.

### 3.3. UV–Vis Spectrometry

The UV–Vis measurements were performed using a spectrophotometer U-2810 from Digilab Hitachi. The experimental conditions for the absorption spectra were: slit width of 1.5 nm, sampling interval of 0.5 nm, and scan speed of 400 nm min-1. All UV–Vis spectra were measured from 230 nm to 400 nm, in a quartz glass cuvette with an optic path of 1 cm. The UV–Vis spectra were recorded, at different incubation times in 0.1 M acetate buffer, pH = 4.5, of solutions of 50 μg mL^−1^ of dsDNA with different concentrations of BTZ. Control solutions of dsDNA or BTZ were also prepared, and UV–Vis spectra were recorded for the same time periods.

### 3.4. Gel Electrophoresis

The electrophoresis gel was prepared from 1% nondenaturing agarose, ultrapure DNA grade from Sigma, in TAE buffer (10 mM Tris base, 4.4 mM acetic acid, and 0.5 mM EDTA, pH = 8.0). A dsDNA control solution of 25 μL and a BTZ–dsDNA sample, with 0.25% bromophenol blue in water, were added into the wells, and electrophoresis was performed in TAE buffer for 2h at ~100 V. Then, DNA was stained with 0.5% ethidium bromide (EtBr) and visualized and recorded under UV at 312 nm transillumination to evaluate DNA mobility. The migration profile was evaluated with ImageJ software, version 1.53t.

### 3.5. Mass Spectrometry

The mass spectra were recorded using an ESI ion source Bruker Daltonik amaZon speed ion trap mass spectrometer (Bruker Daltonik, Bremen, Germany). The ion source was operated in positive mode with nitrogen as the drying gas, with a flow rate of 5 L min^−1^ at 180 °C, a nebulizer pressure of 7.5 psi, a capillary voltage of 4.5 kV, and an end-plate offset of 0.5 kV. Optimum ion transfer was achieved by automatically running the system smart parameter setting in order to optimize the ion transfer for the desired *m*/*z* value. Charge control of the ion trap was activated, with a target value of 200,000 and a maximum accumulation time of 10 ms. Samples of 100 µM BTZ in methanol were injected directly into the mass spectrometer at a flow rate of 180 µL h^−1^. Mass spectra were recorded for *m*/*z* values between 50 and 750 at 5200 a.m.u. s^−1^ in maximum-resolution scan mode, and 10 scans were averaged into one mass spectrum.

The acidic digestion procedure of DNA before and after incubation with BTZ consist in the mechanical removal of the DNA film from the electrode surface. The DNA film, weighing about 350 µg (10 µL drop containing 35 mg mL^−1^ of dsDNA), was treated with HClO_4_ 9 M during 10 min. Then, the digestion was stopped by neutralization with NaOH 9 M. All digested DNA samples were diluted with purified water and methanol and subjected to MS spectrometry.

### 3.6. Viability Assay

L929 cells were grown in Dulbecco’s Modified Eagle Medium (DMEM) culture medium supplemented with 4.5 g L^−1^ of glucose, 2 mM l-glutamine, 10% fetal bovine serum, penicillin (100 U/mL), and streptomycin (100 μg mL^−1^), under controlled conditions (humidity, 5% CO_2_, 37 °C). Sub-cultivation was carried out in cell culture flasks (T-25), and when the cells reached pre-confluence, (~ 80%) they were detached using a trypsin solution (0.25% concentration), counted, and plated for the experimental procedures. Subsequently, the cells were plated in 96-well plates at a density of 10,000 cells/well and placed in the incubator at 37 °C for 24 h prior to the BTZ treatment. After overnight incubation, the medium was changed, BTZ was added at two different concentrations, 1.0 and 5 µg mL^−1^, and the cells were incubated 24 h. Following the incubation, the medium was changed, and 10 µL of the MTS solution was added to each well; after 4 h, the absorption at 490 nm was recorded using the plate reader FLUOstar Omega, BMG Labtech, Ortenberg, Germany.

### 3.7. Incubation Procedures

#### 3.7.1. Procedure 1—Incubation of the Solutions

A solution of 30 µM BTZ and 50 µg mL^−1^ of dsDNA was incubated in 0.1 M acetate buffer, pH = 4.5, during different periods of time. At the same time, control solutions of 50 µg mL^−1^ of dsDNA and 30 µM BTZ were prepared in 0.1M acetate buffer, pH = 4.5, and stored in similar conditions. The DP voltammograms were recorded in solution after different incubation times, always using a clean GCE surface.

#### 3.7.2. Procedure 2—dsDNA Electrochemical Biosensor

The dsDNA electrochemical biosensors were prepared by coating the GCE surface with one 10 μL drop of 35 mg mL^−1^ dsDNA. Then, the electrode surface was dried under a constant flux of N_2_. For the poly[A] and poly[G] electrochemical biosensors, the surface of the GCE was covered with one 10 μL drop of a 150 μg mL^−1^ poly[A] or poly[G] solution, respectively.

The dsDNA- or poly[A] and poly[G] electrochemical biosensors were immersed in solutions of different concentrations of BTZ in 0.1M acetate buffer pH = 4.5, and allowed to incubate during different periods of time, without or with a conditioning applied potential of +0.90 V, as indicated in the text. Afterwards, the biosensors were removed from the solution, washed with deionized water in order to remove the unbounded BTZ molecules, and placed in the electrochemical cell, containing only the 0.1M acetate buffer, pH = 4.5, supporting electrolyte, where the transduction was performed by DP voltammetry.

For the control experiments, the dsDNA electrochemical biosensor was incubated in the 0.1M acetate buffer, pH = 4.5, supporting electrolyte for the same periods and in similar conditions as the solutions of BTZ.

## 4. Conclusions

The interaction between the anti-cancer drug bortezomib and dsDNA was investigated in bulk solutions incubated for different times and at the dsDNA electrochemical biosensor surface.

The dsDNA–bortezomib interaction occurred in one step and involved the electroactive center of bortezomib, leading to small morphological modifications in the DNA double helix, which were electrochemically identified through changes in the guanine and adenine residues oxidation peaks and confirmed by electrophoretic and spectrophotometric measurements.

Controlling the applied potential to the dsDNA electrochemical biosensor surface, the oxidation of the bortezomib amino group in the proximity of the pyrazine ring enabled the in situ electrochemical generation of a bortezomib redox product radical and the study of its interaction with dsDNA. Upon interaction, a decrease in the guanine residue oxidation peak was observed, in agreement with the covalent attachment of the bortezomib redox product to the C8 of the guanine residues, preventing their oxidation. Using polynucleotides of known sequences, it was confirmed that the interaction between bortezomib and dsDNA involved preferentially the guanine-enriched segments. The formation of the bortezomib redox product–guanine adduct was also demonstrated by MS spectrometry.

An interaction mechanism is proposed, and the formation of the bortezomib redox product–guanine adduct explained.

## Data Availability

Data are available upon reasonable request.

## Sample Availability

Samples of the compounds used in this work are available from the producers.
